# Prevalence and factors associated with multidrug/rifampicin resistant tuberculosis among suspected drug resistant tuberculosis patients in Botswana

**DOI:** 10.1186/s12879-019-4375-7

**Published:** 2019-09-06

**Authors:** Blackson Pitolo Tembo, Ntambwe Gustav Malangu

**Affiliations:** 1 National Tuberculosis Reference Laboratory, Ministry of Health, Gaborone, Botswana; 2 Department of Epidemiology and Biostatistics, Sefako Makgatho Health Sciences University, Pretoria, South Africa

**Keywords:** Prevalence, MDR/RR-TB, Associated factors, Retrospective review, Botswana

## Abstract

**Background:**

To investigate the prevalence and factors associated with the prevalence of multidrug/rifampicin-resistant tuberculosis among suspected drug resistant tuberculosis patients in Botswana.

**Methods:**

A retrospective review of medical records of suspected drug resistant tuberculosis patients receiving care at public health facilities in Botswana was conducted from January, 2013 and December, 2014. Patient characteristics and drug susceptibility data were abstracted from 2568 medical records on to a pre-tested checklist form. The prevalence of multidrug/rifampicin resistance was computed. Bivariate and multivariate logistic regression was carried out to determine the factors associated with the prevalence of multidrug/rifampicin in the study population.

**Results:**

Overall, multidrug/ rifampicin - resistance among suspected drug resistant tuberculosis patients in Botswana were found in 139 (5.4%) cases with 1.3% among new cases and 7.7% among previously treated tuberculosis patients. Being a previously treated tuberculosis patient and having a positive smear were found to be factors associated with the prevalence of multidrug/rifampicin-resistant tuberculosis (*p* < 0.05). However, age, sex, living in urban area and HIV status were not associated with this disease (*p* > 0.05).

**Conclusion:**

This study highlights a low burden of multidrug/rifampicin resistant tuberculosis among suspected drug resistant tuberculosis patients receiving care at public health facilities in Botswana. Strategies in controlling MDR/RR-TB should emphasize on effective implementation of Directly Observation Treatment – short course strategy, continuous surveillance of drug resistance cases, prevention of the development of new cases of MDR/RR-TB and to treat existing patients. Further interventions should focus on strengthening TB infection control activities.

## Background

Tuberculosis (TB) is a major public health problem worldwide and the World Health Organization (WHO) estimates that one third of the world’s population approximately 2 billion people are infected with *Mycobacteria tuberculosis* (MTB) the causative agent of TB. In 2015, there were an estimated 10.4 million new incident cases of TB worldwide and an estimated 1.4 million TB deaths occurred [1]. The emergence of drug resistant TB (DR-TB) and in particular multidrug resistant TB (MDR-TB) defined as TB resistant to at least isoniazid and rifampicin and rifampicin resistance - TB (RR-TB) defined as TB resistant to rifampicin only detected using genotypic or phenotypic methods has complicated the management and global control of the disease [2–4].

Globally, it is estimated that 3.9% of all new TB cases and 21% of previously treated TB cases (totaling 580,000 people) developed MDR-TB in 2015 and approximately 250,000 people died from the disease. Three countries namely China, the Russian Federation and India, the most populous countries carry the greatest burden of MDR/RR-TB, together accounting for more than 45% of the world’s total cases. However, countries of the former Soviet Union such as Belarus, Republic of Maldova, Kazakhstan, Azerbaijan, Tajikistan, Kyrgyzstan and Ukraine reported the highest rate of MDR/RR-TB among new TB cases with peaks of up to 37% [1]. Most DR-TB cases in high transmission countries around the world are due to primary infection [1].

Botswana is an upper middle-income country in Southern Africa where TB is highly endemic. Although the TB notification rate in the country has decreased from 623 per 100,000 population in 2002 to 305 per 100,000 population in 2014, it still remains one of the highest in the world [5]. Mortality rates have been reported to be as high as 13% of all adult cases and 40% among people living with HIV/AIDS [6, 7]. So far four drug resistant surveys (DRS) performed to establish the level of drug resistance in Botswana showed that anti-TB drug resistance was a growing problem. The first DRS conducted in 1996–1997 revealed a low prevalence of MDR-TB estimated at 0.2% among new TB cases and 6.1% among previously treated TB patients and since then it has shown a progressive increase over the years. The 4th and most recent country-wide survey conducted in 2008 showed that MDR-TB was 2.5% among new cases and 5.5% among previously treated TB cases [6, 8, 9].

Factors such as non-adherence to prescribed medication by the patient, physician error associated with inadequate or inappropriate chemotherapy prescribed, and poorly functioning National Tuberculosis Programme associated with poor drug quality, lack of Directly Observed Treatment Short-course (DOTS) and irregular drug supply have been associated with the prevalence of MDR/RR-TB in many settings [10–12]. However, there is no information on the role of these factors in the prevalence of MDR/RR-TB in Botswana. Understanding factors associated with the prevalence of MDR/RR-TB in Botswana is critical to reducing the burden, to decide on health priorities and to allocate resources [13]. Therefore, the purpose of this study was conducted to determine the current burden and factors associated with the prevalence of MDR/RR-TB among suspected DR-TB patients in Botswana which could be used to design an effective control programme.

## Methods

### Study design and study population

This study was based on a retrospective review of medical records of suspected DR-TB patients receiving care at 33 public health facilities in Botswana between January, 2013 and December, 2014. Health care system in Botswana is comprised of 26 health districts and is based on the primary health care approach. The system is highly decentralized and TB services including physical examination, smear microscopy, culture and chest X-Ray are delivered by trained health care workers and volunteers through a network of hospitals, clinics, health posts and mobile health facilities [6, 14].

Botswana’s guidelines for DR-TB follow the WHO recommendations with the diagnosis supported by decentralized molecular drug susceptibility testing (DST) using Xpert MTB/RIF (Gene Xpert) and Centralized phenotypic DST performed at the National Tuberculosis Reference Laboratory (NTRL) using the conventional Lowenstein-Jensen Proportion Method (LJ) [6, 14, 15]. Only patients considered to be at high risk of DR-TB such as MDR-TB contacts, patients lost to follow-up, relapsing cases and treatment failures were tested for DR-TB. New cases were subjected for DST using Gene Xpert while previously treated cases were tested using the conventional DST Lowenstein-Jensen proportion method.

In line with the national routine HIV testing policy, all persons with signs and symptoms of TB were offered the HIV test [14]. TB treatment was free of charge and was provided under direct observation by a DOTS provider [6, 8, 15]. Patient information within public health facilities was managed using multiple systems including the Integrated Patient Management System (IPMS), Electronic TB Registers (ETR), DISA Laboratory Information Systems and Paper-based registers that enabled availability of information on all TB patients [6, 8, 14, 15].

### Study population

**Fig. 1:**
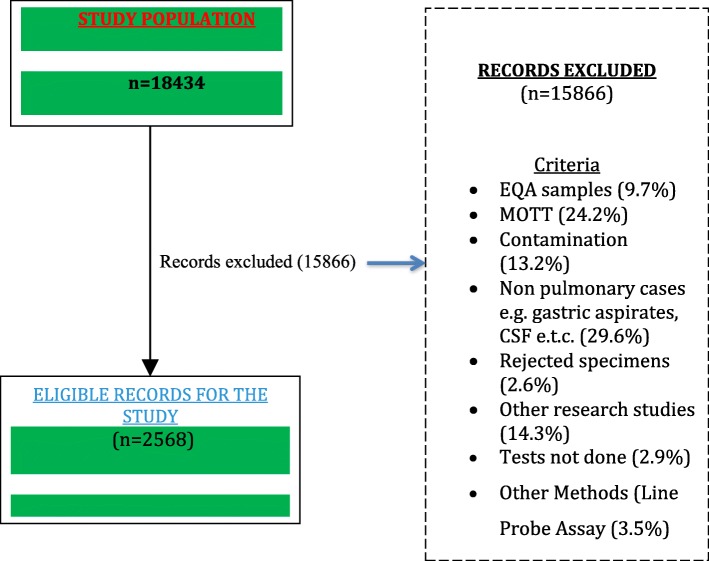
Enrollment of the study population

Suspected DR-TB patients receiving care at public health facilities in Botswana constituted the study population. No sampling was done in this study; instead a census was conducted on all eligible TB cases. All new and previously treated TB cases with pulmonary disease, both male and female patients of all age groups with DST results from LJ or Gene Xpert platform formed the inclusion criteria while all cases of extra-pulmonary TB, patients without DST results, patient without patient category and patients seeking TB care at a private health care facility formed the exclusion criteria (Fig. 1). In addition, all cases that showed discordant results between LJ and Gene Xpert were also excluded from the study.

### Data collection

A total of 15,866 TB records were reviewed during the study period and 2568 eligible cases were extracted and enrolled in the study. Relevant patient demographic information (age, sex, patient residence (urban/peri-urban, versus rural), clinical data (HIV status, smear status, category of the patient), and laboratory data (test method employed for drug resistance and DST test results) were abstracted at baseline from laboratory registers, treatment registers, database at NTRL database and from Botswana National Tuberculosis Programme (BNTP) electronic TB register. A pre-tested standardized checklist form specifically designed for this study was used to collect data from the reviewed medical records.

### Operational definitions

The following definitions related to drug resistance and patient category were used [16–18].

- MDR-TB was defined as the in vitro resistance to at least both isoniazid and rifampicin.
- Rifampicin resistance was defined as in vitro resistance to rifampicin only – a surrogate marker of MDR-TB.
- A new case was defined as a patient who had never had treatment for TB before or had taken TB treatment for less than 1 month.
- Previously treated TB case was defined as a patient who had previously taken TB treatment for more than 1 month and included relapses, treatment failure, and treatment after default also known as lost to follow-up.
- Suspected DR-TB patient refers to a TB patient who has previously been treated for TB before such as relapsing cases, lost to follow-up and treatment failure or a TB patient infected with a DR-TB strain.

### Outcome of interest

This review was intended to measure the prevalence of MDR/RR-TB among suspected DR-TB patients receiving care at a public health facility in Botswana. MDR/RR-TB prevalence was calculated for the total sample and stratified for new and previously treated TB patients.

### Data analysis

Data were double entered in Excel spread sheet to ensure accuracy and exported into Statistical Package for Social Sciences (SPSS) software, (version 21, SPSS, Inc. Chicago, Illinois, USA) for analysis. The proportion of MDR/RR-TB among the study population was calculated. Bivariate analysis was carried out to test the association between the dependent variable MDR/RR-TB and various demographic and clinical factors**.** All factors which were significant in the bivariate analysis were entered in the multivariate logistic regression analysis and results were expressed as odds ratios (OR) with 95% confidence Interval (CI). For all statistical analysis, a *p* value of < 0.05 was considered significant.

### Ethical consideration

The study was conducted using routinely collected patient data from the patients medical records kept at public health facilities. The retrospective nature of this study meant that informed consent from the patients was not necessary. However, ethical clearance was obtained from the Medical Research Ethics Committee (MREC) of the Sefako Makgatho Health Sciences University, Pretoria, South Africa (Reference number: MREC/H/21/2013: PG) and permission to conduct the study in Botswana was granted by the Health Development Research Committee of the Ministry of Health (Reference number PPME - 13/18/1 Vol V111 (269). Further permission to access patient’s medical records was obtained from Institutional Review Boards (IRBs) of study sites or directly from Chief Executive Officers.

## Results

A total of 2568 TB patients receiving care at public health facilities in Botswana and meeting the inclusion criteria were enrolled in this retrospective study. These included 917 (35.7%) new TB cases and 1651 (64.3%) of previously treated TB cases. The sample was predominantly male accounting for 1317 (51.3%) while female cases constituted 1159 (45.1%), giving a male/female ratio of 1.14: 1. Most cases were adult patients with a mean age of 40.8 years (range 1 to 88 years. Of the cases included in the study, the majority of the patients 1471 (57.3%) resided in urban/peri-urban areas of the country. Overall HIV prevalence in the study population was 55.5% (Table 1).

**Table 1:** Socio-demographic characteristics of the study population (*n* = 2568)

Variable	Frequency
Age group (in years)	
< 14 years	87 (3.4%)
> 14 years	2083 (81.1%)
Unknown	398 (15.5%)
Gender	
Male	1317 (51.3%)
Female	1159 (45.1%)
Unknown	92 (3.6%)
HIV status	
Positive	1426 (55.5%)
Negative	394 (15.3%)
Unknown	748 (29.1%)
Smear status	
Positive	497 (19.3%)
Negative	1273 (49.6%)
Unknown	798 (31.1%)
Patient residence	
Urban/peri-urban	1471 (57.3%)
Rural	1090 (42.4%)
Other	7 (0.3%)
Patient Category	
New TB cases	917 (35.7%)
Previously treated TB cases	1651 (64.3%)
DST method	
Gene Xpert	863 (33.6%)
LJPM	1705 (66.4%)

### Prevalence of multidrug/rifampicin resistant tuberculosis among suspected DR-TB patients in Botswana

**Fig. 2:**
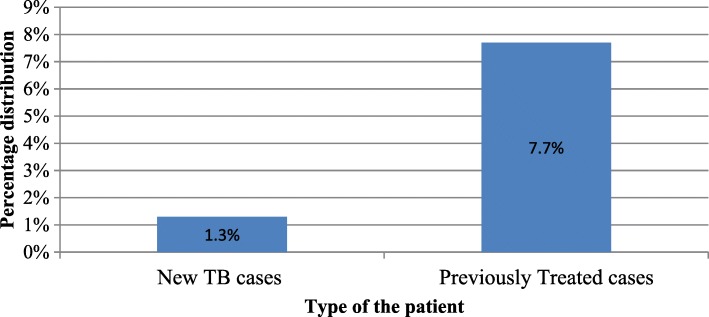
Proportion of MDR/RR-TB among new and previously treated TB cases

A total of 2568 medical records of suspected DR-TB patients who tested for drug resistance using Gene Xpert and conventional LJPM were included in this retrospective study. Overall, MDR/RR-TB was reported in 139 cases (5.4%) which included 1.3% among new TB cases and 7.7% among previously treated TB cases (Fig. 2).

**Fig. 3:**
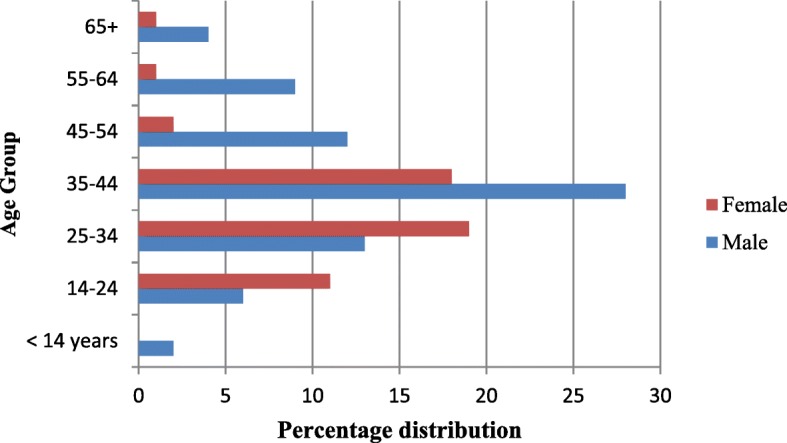
Distribution of multidrug-resistant tuberculosis cases among suspected DR-TB patients stratified by age group and sex

Figure 3 below shows the distribution of MDR/RR-TB by gender and age group. The highest proportion of MDR/RR-TB was among the male population in the 35–44 years age group.

### Prevalence of multidrug/rifampicin resistance among suspected DR-TB patients stratified by method of the test used

**Fig. 4:**
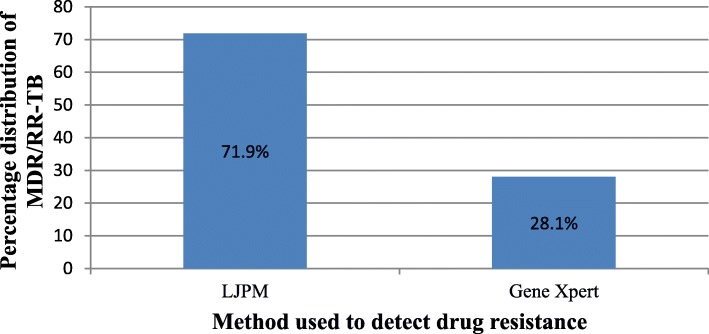
Methods used to detect drug resistance

Figure 4 shows the distribution of MDR/RR-TB by method of the test. From 139 cases of MDR/RR-TB identified during the review, 100 (71.9%) cases were detected by the conventional DST using LJ while 39 (28.1%) were detected using Gene Xpert molecular technology.

### Factors for the prevalence of multidrug/rifampicin resistant TB among suspected DR-TB patients in Botswana

Table 2 below presents factors associated with the prevalence of MDR/RR-TB among suspected DR-TB patients in Botswana. Socio-demographic determinants such as age, sex, place of residence of the patient, category of the patient, HIV status, and smear status were all assessed for association with the prevalence of MDR/RR-TB. In bivariate analysis, a patient who suffered from MDR/RR-TB was more likely to be an urban dweller (*p* < 0.008), Smear positive (*p* < 0.001), HIV positive (*p* = 0.005), and a previously treated TB case (*p* < 0.001). Sex and age were not found to be associated with the prevalence of multidrug/rifampicin resistance.

**Table 2:** Multivariate logistic regression of factors associated with the prevalence of MDR/RR-TB among suspected DR-TB patients

Factor	OR (95% CI)	*p*-value
HIV positive cases versus HIV negative cases	759 (.477–1.208)	.245
Smear positive cases versus smear negative cases	20.130 (11.916–33.860)	< 0.001
Urban population versus rural population	758 (.480–1.197)	.234
Previously treated TB cases versus new TB cases	4.468 (2.095–9.529)	< 0.001

*OR* Odds Ratio, *HIV* Human Immunodeficiency Virus, *CI* Confidence Interval

However, in a multivariate logistic regression analysis, only history of previous TB treatment, and being smear positive were both found to be associated with the prevalence of MDR/RR-TB in this study (*p* < 0.05), while HIV and living in urban area were not found to be associated with the prevalence of the disease (*p* > 0.05) (Table 2).

## Discussion

Botswana has one of the highest TB burden in the world estimated to be as high as 305 cases per 100,000 population and has also documented a growing increase in DR-TB and in particular MDR-TB in previous national DRS [6, 7]. Prompt and appropriate management of MDR/RR-TB cases, including strictly adherence to therapy is required to achieve control over the disease [19]. This study assessed the current burden and factors associated with the prevalence of MDR/RR-TB among suspected DR-TB patients in Botswana using data collected from public health facilities from January, 2013 and December, 2014.

Overall the rate of MDR/RR-TB was 5.4% in the study population with 1.3% among new TB cases and 7.7% among previously treated TB patients. This finding is consistent with the 2015 and 2016 WHO Global TB reports which have documented low levels of MDR/RR-TB (< 3%) among new TB cases in many parts of the world [1, 19, 20]. In sharp contrast, other settings in the WHO African region have reported higher rates of MDR/RR-TB. The rate of MDR/RR-TB among new and previously treated TB cases in Nigeria, Democratic Republic of Congo, Ethiopia, South Africa and Somalia are 4.3 and 25%; 3.2 and 14%; 2.7 and 14%; 3.5 and 7.1%; 8.7 and 47% respectively [1]. The difference in the findings between the new cases and previously treated TB cases in this study might be attributed to the difference in proportion of new cases and previously treated cases in the sample since the majority of the cases in the study were previously treated TB cases. The results also reflect that previously treated TB patients were more likely to harbor DR-TB than new cases.

Previous Ethiopian studies have reported that inadequate treatment regimen prescribed by health staff, poor patient adherence, previous history of exposure to anti-TB drugs were common factors for the prevalence of TB drug resistance [10, 20–22] Other studies elsewhere have also reported younger age, urban residence, non-permanent residents, known TB contact, rural population, HIV infection and female sex [21] as the most common factors associated with the prevalence of drug resistance. In this study a history of previous TB treatment and being smear positive were found to be associated with the prevalence MDR/RR-TB (*p* < 0.05). However, our study did not observe any statistically significant association between the prevalence of MDR/RR-TB and certain patient characteristics such as age, gender, HIV status and population type (*p* > 0.05).

The current study also showed that patient category was associated with the prevalence of MDR/RR-TB. Being a previously treated TB case was associated with prevalence of MDR/RR-TB 6.278 times compared with being new a TB case. This finding is consistent with many studies conducted elsewhere that indicated that a history of previous exposure to anti-TB treatment was the most significant factor associated with the prevalence of MDR/RR-TB [2, 3, 11, 23, 24]. This finding may be related to unsatisfactory compliance by patients or clinicians, lack of treatment supervision, improper drug regimens and inadequate or irregular drug supply that make the bacteria to mutate and develop drug resistance [2, 10].

In this study smear status was also associated with the prevalence of MDR/RR-TB. Smear positive patients were 17 times more likely to have MDR/RR-TB compared to patients who were smear negative. This finding is in agreement with other studies conducted in Thailand, Iran and Malaysia [25, 26]. One possible explanation is that smear positivity at the end of the treatment period could be the result of drug resistance rather than the cause. Age and gender were not associated with the prevalence of MDR/RR-TB in this study compared with studies conducted elsewhere which found that age and gender were important factors associated with MDR/RR-TB [23, 27].

A study in Pakistan reported that early age (between 10 and 25 years) was a strong risk factor for the development of MDR-TB [27]. However, a Malaysian study did not find any significant association between the development of MDR-TB and age [23]. These conflicting results observed shows that there is no well-established association between age and the prevalence of MDR-TB because different studies used different age group cut-off points compared to this study which compared younger patients less than 14 years to patients 14 years and older.

Based on the data obtained, early case detection and prompt initiation of appropriate therapy is required to interrupt further transmission. Targeted policies for previously treated TB patients and smear positive cases will significantly reduce the burden of the disease including the implementation of a high quality DOTS program involving supervision and follow-up of patients taking their medication. Furthermore, there is need to strengthen drug resistance surveillance monitoring systems and the implementation of effective infection control measures in order to reduce the burden of MDR/RR-TB.

This study, however had its own limitations. First, the study was performed retrospectively and some of data was found to be missing including patients characteristics such as age, sex, HIV status and drug susceptibility testing results due to poor documentation. Secondly the current data were only collected from suspected DR-TB patients and so may not reflect all TB cases. Thirdly factors associated with the prevalence of MDR/RR-TB were limited only to age, sex, HIV status, smear status, population type and patient category. Fourthly, the results of this study can only be generalized to a high risk group of DR-TB. Despite these limitations, the study has provided useful information with regards the current burden and the factors associated with the prevalence of MDR/RR-TB in Botswana which can be used for better planning of TB management in the country.

## Conclusion

This study has revealed important information on the current prevalence and factors associated with the prevalence of MDR/RR-TB in Botswana. Based on the results obtained, this study has demonstrated low levels of MDR/RR–TB in Botswana. History of previously anti-TB treatment and a positive smear were the only statistically significant factors associated with the prevalence of MDR/RR-TB. Therefore, strategies in controlling MDR/RR-TB should emphasize on effective implementation of DOTS strategy, continuous surveillance of drug resistance, prevent the development of new cases of MDR/RR-TB and to treat existing patients. Further interventions should focus on strengthening TB infection control activities.

## Data Availability

The datasets used and/or analyzed during the current study are available from the corresponding author on reasonable request.

## Abbreviations

BNTP: Botswana National Tuberculosis Programme
DOTS: Directly Observed Treatment-short course
DRS: Drug Resistant Survey
DR-TB: Drug Resistant Tuberculosis
DST: Drug Susceptibility Testing
HIV: Human Immunodeficiency Virus
IRB: Institutional Review Board
LJPM: Lowenstein-Jensen Proportion Method
MDR-TB: Multidrug Resistant Tuberculosis
MREC: Medical Research Ethics Committee
RR-TB: Rifampicin Resistant TB
TB: Tuberculosis
WHO: World Health Organization

## References

[1] World Health Organization. Global Tuberculosis Report 2016. World Health Organization, 2016. WHO/HTM/TB/2016.13.

[2] Desissa F, Workineh T, Beyene T (2018). Risk factors for the occurrence of multidrug-resistant tuberculosis among patients undergoing multidrug – resistant tuberculosis treatment in east Shoa, Ethiopia. BMC Public Health.

[3] Daniel Olusoji, Osman Eltayeb (2011). Prevalence and risk factors associated with drug resistant TB in South West, Nigeria. Asian Pacific Journal of Tropical Medicine.

[4] Girum T, Tariku Y, Dessu S (2017). Survival status and treatment outcome of multidrug resistant tuberculosis (MDR-TB) among patients treated in treatment initiation centers (TIC) in South Ethiopia: a retrospective cohort study. Ann Med Health Sci res.

[5] Barman N, Ghosh D, Rahman Q, Uddin MN, Ahmed S, Paul D (2014). Assessment of risk factors of multidrug resistant tuberculosis with emphasis on serum zinc. Bangladesh Med J.

[6] Botswana Ministry of Health. National Tuberculosis Control Program Combined Annual Report 2013 - 2014. Ministry of Health, Republic of Botswana, Gaborone, pp22.

[7] Auld AF, Agizew T, Pals S, Finlay A, Ndwapi N, Boyd R (2016). Implementation of a programmatic, stepped-wedge cluster randomized trial to evaluate impact of Botswanaʼ s Xpert MTB/RIF diagnostic algorithm on TB diagnostic sensitivity and early anti-retroviral therapy mortality. BMC Infect Dis.

[8] Botswana Ministry of Health. National Tuberculosis Control Program Strategic Plan 2013–2017. pp, 3–11, Gaborone, Ministry of Health; 2013.

[9] Menzies HJ, Moalosi G, Anisimova V, Gammino V, Sentle C, Bachhuber MA (2014). Increase in anti-tuberculosis drug resistance in Botswana: results from the fourth National Drug Resistance Survey. Int J Tuberc Lung Dis.

[10] Mulu W, Mekonnen D, Yimer M, Admassu A, Abera B (2015). Risk factors for multidrug resistant tuberculosis patients in Amhara National Regional State. Afr Health Sci.

[11] Baghaei P, Tabarsi P, Chitsaz E, Novin A, Alipanah N, Kazempour M, Mansouri D (2009). Risk factors associated with multidrug-resistant tuberculosis. Tanaffos.

[12] Franden G, Pennington SS. Abramsʼ clinical drug therapy: rationales for nursing practice. 10^th^ edition. Philadelphia: Wolter Kluver Health and Lippincott Williams and Wilkins; 2014. p. 351.

[13] Mekonnen F, Tessema B, Moges F, Gelaw A, Eshetie S, Kumera G (2015). Multidrug resistant tuberculosis: prevalence and risk factors in districts of Metema and west Armachiho, Northwest Ethiopia. BMC Infect Dis.

[14] Botswana Ministry of Health. Botswana National Tuberculosis Programme Manual 6th edition. Gaborone, ministry of health, 2007.

[15] Botswana Ministry of Health. National Tuberculosis Control Program Strategic Plan 2013–2017. Gaborone, Ministry of Health; 2013.

[16] World Health Organization. Guidelines for the programmatic management of drug-resistant tuberculosis. WHO 2006: WHO/HTM/TB/2006.361. 23844450

[17] Sengul A, Akturk UA, Aydemir Y, Kaya N, Kocak ND, Tasolar FT (2015). Factors affecting successful treatment outcomes in pulmonary tuberculosis: a single-center experience in Turkey, 2005-2011. J Infect Dev Ctries.

[18] World Health Organization. Definitions and reporting framework for tuberculosis – 2013 revision. Geneva, Switzerland. WHO/HTM/TB/2013.2.

[19] World Health Organization. Global Tuberculosis Report 2015. World Health Organization, 2016. WHO/HTM/TB/2015.22.

[20] Eshetie S, Gizachew M, Dagnew M, Kumera G, Woldie H, Ambaw F, Tessema B, Moges F (2017). Multidrug resistant tuberculosis in Ethiopian settings and its association with previous history of anti-tuberculosis treatment: a systematic review and meta-analysis. BMC Infect Dis.

[21] Mulisa G, Workneh T, Hordofa N, Suaudi M, Abebe G, Jarso G (2015). Multidrug-resistant *Mycobacterium tuberculosis* and associated risk factors in Oromia region of Ethiopia. Int J Infect Dis.

[22] Berhan A, Berhan Y, Yizengaw D (2013). A meta-analysis of drug resistant tuberculosis in sub-Saharan Africa: how strongly associated with previously treatment and HIV co-infection. Ethiop J Health Sci.

[23] Lomtadze N, Aspindzelashvili R, Janjgava M, Mirtskhulava V, Wright A, Blumberg HM (2009). Prevalence and risk factors for multidrug-resistant tuberculosis in the republic of Georgia: a population-based study. Int J Tuberc Lung Dis.

[24] Jimma W, Ghazisaeedi M, Shahmoradi L, Abdurahman AA, Kalhori SRN, Nasehi M, Yazdi S, Safdari R. Prevalence of and risk factors for multidrug-resistant tuberculosis in Iran and its neighboring countries: systematic review and meta-analysis. Rev Soc Bras Med Trop vol.50 no.3 Uberaba May/June 2017. 10.1590/0037-8682-0002-2017 28700044

[25] Chuchottaworn C, Thanachartwet V, Sangsayunh P, Than TZM, Sahassananda D, Surabotsophon M, Desakom V (2015). Risk factors for multidrug-resistant tuberculosis among patients with pulmonary tuberculosis at the central chest Institute of Thailand. PLoS One.

[26] Farazi A, Sofian M, Zarrinfar N, Katebi F, Hoseini SD, Keshavarz R (2013). Drug resistance pattern and associated risk factors of tuberculosis patients in the central province of Iran. Caspian J Intern Med.

[27] Ullah I, Javaid A, Tahir Z, Ullah O, Shah AA, Hasan F (2016). Pattern of drug resistance and risk factors associated with development of drug resistant *Mycobacterium tuberculosis* in Pakistan. PLoS One.

